# Elevation of soluble interleukin-2 receptor levels in nasal allergy

**DOI:** 10.1155/S096293519500007X

**Published:** 1995-01

**Authors:** K. Hisamatsu, T. Ganbo, T. Nakazawa, S. Horiguchi, S. Shimomura, Y. Murakami

**Affiliations:** Department of Otorhinolaryngology Yamanashi Medical University 1110 Shimokato Tamaho-cho, Nakakoma-gun, Yamanashi 409-38 Japan

## Abstract

To investigate soluble IL-2 receptor (sIL-2R) levels in nasal allergy, the sera and nasal secretions from patients with nasal allergy and from healthy subjects were subjected to a double-epitope enzyme-linked immunosorbent assay. Significant elevation of sIL-2R concentrations in the sera and nasal secretions was observed in the allergy patients (n = 26) compared with those of healthy subjects (n = 9). IL-2R-positive (CD25^+^) cells were observed in the crust formed in an allergic nasal mucosa. The concentration of sIL-2R in the sera correlated neither with the eosinophil count of the peripheral blood count nor with clinical severity. The concentration of sIL-2R in the nasal secretions was significantly higher compared with that in the sera from allergic patients (p < 0.01), whereas no significant difference was observed between sIL-2R levels in the sera and nasal sections from normal subjects. These findings indicate that sIL-2R plays an essential role in allergic processes by regulating IL-2R-positive cells recruited into the nasal mucosa.


Mediators of Inflammation 4, 39–42 (1995)

### Elevation of soluble interleukin-2 receptor levels in nasal allergy

K. Hisamatsu,[CA] T. Ganbo, T. Nakazawa, S. Horiguchi, S. Shimomura and Y. Murakami

Department of Otorhinolaryngology, Yamanashi Medical University, 1110 Shimokato, Tamaho-cho, Nakakoma-gun, Yamanashi 409-38, Japan

[CA] Corresponding Author

**To investigate soluble IL-2 receptor (sIL-2R) levels in nasal allergy, the sera and nasal secretions from patients with nasal allergy and from healthy subjects were subjected to a double-epitope enzyme-linked immunosorbent assay. Significant elevation of sIL-2R concentrations in the sera and nasal secretions was observed in the allergy patients ($n = 26$) compared with those of healthy subjects ($n = 9$). IL-2R-positive ($CD25^+$) cells were observed in the crust formed in an allergic nasal mucosa. The concentration of sIL-2R in the sera correlated neither with the eosinophil count of the peripheral blood count nor with clinical severity. The concentration of sIL-2R in the nasal secretions was significantly higher compared with that in the sera from allergic patients ($p < 0.01$), whereas no significant difference was observed between sIL-2R levels in the sera and nasal sections from normal subjects. These findings indicate that sIL-2R plays an essential role in allergic processes by regulating IL-2R-positive cells recruited into the nasal mucosa.**

**Key words:** CD25, Nasal allergy, Nasal secretion, Soluble interleukin-2 receptor

### Introduction

Nasal allergy is a typical IgE-mediated allergic inflammation of the nasal mucosa where recruitment of eosinophils, lymphocytes and mast cells, is observed. Recent studies have elucidated the role of mast cells and eosinophils in nasal allergy. However, the role of lymphocytes in this condition remains to be determined despite advances in immunological studies of cytokines and their receptors. Studies on the role of lymphocytes in nasal allergy is thus crucial for elucidation of the mechanism of allergic inflammation in the nose, since lymphocyte activation following signalling by antigen-presenting cells may initiate subsequent sequential immunological events.

Soluble IL-2 receptor (sIL-2R) is an extracytoplasmic component of the α chain subunit shed from the IL-2 receptor. Recently, the elevation of serum sIL-2R level has been reported in some diseases, including bronchial asthma. However, few studies have been performed on sIL-2R level in nasal allergy. In the present study, sIL-2R levels were determined in the sera and nasal secretions obtained from nasal allergy patients and from healthy control subjects.

### Materials and Methods

*Subjects:* Twenty-six patients were diagnosed as having nasal allergy to house dust mite according to the following criteria: (1) nasal symptoms, namely persistent and recurrent nasal symptoms consisting of sneezing attacks, watery nasal discharge and nasal obstruction; (2) eosinophilia in nasal smear test; (3) positive intradermal allergen reaction; and (4) positive serum IgE antibody which is specific for dermatophagoides pteronyssinus, but not for pollens from trees or grass, or fungus allergens. All patients were free from other infectious and inflammatory diseases for 4 weeks, and medications were discontinued for 2 weeks before the allergological tests and collection of nasal lavage fluids. Nine normal subjects, age- and sex-matched and negative in all these allergy tests, volunteered to act as controls. The age of the patients ranged from 21 to 32 years, and the normal subjects ranged from 23 to 30 years in age. Nasal symptom scores were assessed from a symptom diary and evaluated to determine severity.

*Preparation for the measurement of sIL-2R:* Sera and nasal lavage fluids were collected from 26 patients with perennial nasal allergy and from nine normal subjects. Peripheral blood was extracted and immediately centrifuged twice at 1350 × **g** for 10 min at 4°C, and the resultant sera were stored at –80°C until sIL-2R level was measured.

Nasal smears were obtained prior to nasal lavage and investigated cytologically by Hansel's staining technique,[1] and nasal cytograms were graded semiquantitatively.[2]

© 1995 Rapid Communications of Oxford Ltd

Nasal lavage was performed with 20 ml of 0.9% saline solution with 1 mM LiCl pre-warmed to 37°C using a plastic syringe, and the total volume of recovered lavage fluid was measured. Suputolidin® (Boehringer Diagnostics, La Jolla, CA, USA) was mixed with nasal lavage fluid to homogenize at a volume/concentration ratio of 0.4/9.0 and immediately vortexed for 1 min, after which 5.5% Aprotinin (Sigma, MO, USA) was added at a volume/concentration ratio of 0.1/0.94, followed by immediate mixing for 1 min at room temperature. The mixture was allowed to stand for 30 min at room temperature, then centrifuged at 1 350 × **g** twice each time for 10 min at 4°C, and the supernatant was stored at −80°C until sIL-2R was assayed in duplicate. The lithium (Li) concentration of the nasal lavage fluid was measured by atomic emission spectrophotometry to calculate sIL-2R concentration in the nasal secretions. Li was used as an exogenous marker of nasal secretion allowing measurement from small amounts of nasal secretion.[3] The sIL-2R concentration of the nasal secretion (sIL-2R*x*) was calculated from the following equation: sIL-2R*x* = sIL-2Rn × Lio/(Lio–Lin), where sIL-2Rn denotes the sIL-2R concentration of the sample; Lio, the Li concentration of the lavage fluid containing 0.9% NaCl; and Lin, the Li concentration of the sample.

*Measurement of sIL-2R by enzyme-linked immunosorbent assay:* The double-epitope enzyme-linked immunosorbent assay was used for detecting sIL-2R in the sera and nasal lavage fluids (Cellfree Interleukin-2 Receptor Bead Assay Kit®, T-cell Science, MA, USA). For the measurement of sIL-2R in the nasal lavage fluids, the effect of the lavage fluid itself on the sIL-2R assay was preliminarily tested and no significant effect was found. Briefly, three nasal lavage fluid sampleswere used for the test. An equal volume of standard sIL-2R was added to each nasal lavage fluid and the actual increase in sIL-2R concentration was compared with the calculated value. The mean (±S.D.) actual increase was 95±4% of the calculated value.

The assay was performed in duplicate. Briefly, 50 μl of standard or appropriately diluted serum was added to 150 μl of the horseradish peroxidase-conjugated mouse anti-human sIL-2R monoclonal antibody. One polystyrene bead coated with anti-human sIL-2R monoclonal antibody was incubated in the above mixture for 90 min at room temperature on a rotator set at 150 rpm. The bead was then washed three times with 2 ml of deionized water each time. Two hundred μl of the substrate (*o*-phenylenediamine) was added to each tube and incubated with the bead for 30 min at room temperature, and then 1 μl of $H_2SO_4$ was added to stop the reaction. The solution was then decanted into a 96-well flat-bottomed plate for application to an automatic spectrophotometer (Titerteck Multiscan® Plus MKII, Flow Laboratories Inc., CA, USA). The absorbance of the samples was measured with the spectrophotometer after setting the zero using the substrate blank. IL-2R concentration in the nasal secretion was calculated according to the above equation.

*Immunocytochemical staining for CD25⁺ cells:* Specimens of the inferior turbinate mucosa were fixed in 4% paraformaldehyde, embedded in OCT compound and frozen at −80°C. Fifteen μm-thick sections were cut, immersed in PBS containing 0.3% Triton-X, and incubated in methyl alcohol containing 0.3% $H_2O_2$ for 20 min to inhibit endogenous tissue peroxidase activity. After washing in PBS, the sections were further incubated in normal goat serum for 30 min, washed in PBS again, and then incubated overnight with mouse monoclonal anti-human CD25 antibody (Dako Ltd, High Wycombe, Buckinghamshire, UK), at a dilution of 1:400. The sections were then washed in PBS, and incubated with the biotinylated goat anti-mouse IgG (the second antibody, Nichirei, Tokyo, Japan) for 30 min, washed in PBS again, and incubated with peroxidase-conjugated streptavidin for 30 min. The reaction was then developed by treatment with diaminobenzidine for 10 min followed by washing in PBS. The sections were subsequently fixed in osmic acid solution and dehydrated before mounting.

*Statistics:* The Kruskal–Wallis test was used to evaluate the relationship between sIL-2R values and clinical severity. To evaluate the differences between the values of the two groups, Wilcoxon rank-sum test was used.

### Results

*Levels of sIL-2R in the sera:* The mean (± S.D.) values of serum sIL-2R concentrations in the allergic patients and of the normal subjects were 513 ± 171 U/ml and 310 ± 137 U/ml, respectively. In patients with nasal allergy, the serum sIL-2R concentration was significantly higher as compared with that of the normal subjects ($p < 0.01$, Fig. 1). However, there was no significant correlation between serum sIL-2R concentration and clinical severity.

*Levels of sIL-2R in nasal secretions:* The mean (± S.D.) values of sIL-2R concentrations in the nasal secretions of the patients and of the normal subjects were 1 513 ± 1 233 U/ml and 371 ± 193 U/ml, respectively. The concentration of sIL-2R was significantly higher in the nasal secretions of nasal allergy patients compared with that of the normal subjects ($p < 0.01$). However, there was no significant correlation between the concentrations of sIL-2R in the nasal secretions and clinical severity (Fig. 2). The sIL-2R level

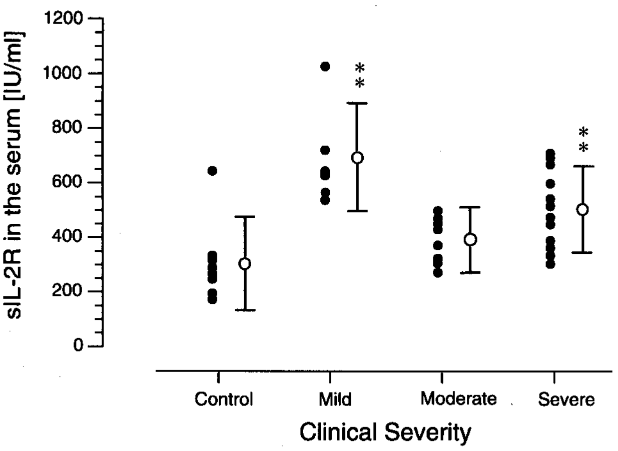

FIG. 1. Levels of serum sIL-2R and clinical severity. Elevation of serum sIL-2R level was observed in nasal allergy patients ($n$ = 26) compared with normal subjects ($n$ = 8, $^{**}p < 0.01$), although there was no significant correlation between serum sIL-2R level and clinical severity.

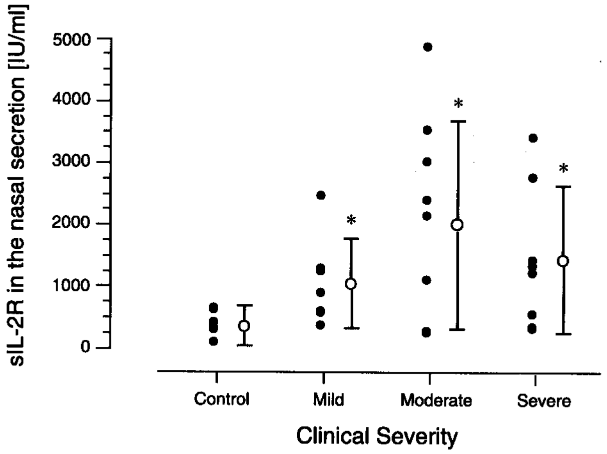

FIG. 2. Levels of sIL-2R in the nasal secretions and clinical severity. The sIL-2R level of the nasal secretions was elevated in the nasal allergy patients ($n$ = 26) compared with the normal subjects ($n$ = 9) ($^{*}p < 0.05$). There was no significant correlation between sIL-2R concentration in the nasal secretions and clinical severity.

detected in the nasal secretions was significantly higher than that in the serum ($p < 0.01$).

*Presence of CD25⁺ cells:* CD25⁺ cells were found in the nasal mucosa from the inferior turbinate of nasal allergy patients (Fig. 3).

### Discussion

Interleukin-2 (IL-2) is a T cell growth factor and plays a central role in the regulation of immune responses. IL-2 is released from T cells activated by IL-1 and antigen or mitogen.[4] Cells responsive to IL-2 include T cells, B cells, natural killer cells and macrophages/monocytes. Recent studies have shown that IL-2R has three subunits, an α chain,[5] a β chain,[6] and a γ chain[7]. The α chain has a low affinity, while the combination of the β and γ chains shows a moderate affinity for IL-2. These three submits combine to form a high affinity IL-2R, and binding of IL-2 to IL-2R expressed on the cell membrane induces cellular activation, differentiation and proliferation. Intracellular signalling through IL-2R is mediated via the associated β and γ subunits. The sIL-2R is found in the T cell culture medium containing a mitogen,[8] and in the serum of patients suffering from diseases such as adult T cell leukaemia,[9] Kawasaki disease,[10] rheumatoid arthritis,[11] and bronchial asthma.[12] In general, T cells are activated through the IL-2/IL-2R system and sIL-2R may inhibit the response of the IL-2-sensitive cells to IL-2.[11] However, the role of sIL-2R *in vivo* still remains unclear.

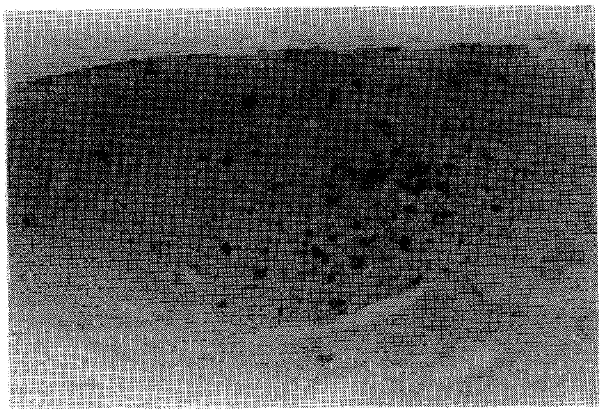

FIG. 3. Immunostaining of the nasal mucosa obtained from the inferior turbinate of a nasal allergy patient for CD25 (IL-2R). CD25⁺ cells formed a cluster in superficial lamina propria. (Original magnification × 185.)

In the present study, we demonstrated elevation of sIL-2R concentrations in both serum and nasal secretions, and observed infiltration of CD25⁺ cells in the nasal mucosa of nasal allergy patients immunocytochemically. Since CD25⁺ cells are not confined only to T cells but also include B cells, natural killer cells and macrophages/monocytes, sIL-2R may originate from all these cells. Thus, further studies are required to determine the cellular origin of sIL-2R in nasal allergy.

Regarding the origin of sIL-2R, an immunohistological study of the allergic nasal mucosa after exposure to allergens demonstrated a significant increase in the number of CD25⁺ (IL-2R-positive) cells and CD4⁺ helper T cells, and showed a significant correlation between CD4⁺ and CD25⁺ cells but not between macrophages and CD25⁺ cells. There were no changes in distribution of CD45⁺ cells, CD3⁺ cells or CD8⁺ cells in the nasal mucosa. A significant increase in the ratio of CD4⁺/CD8⁺ lymphocytes was observed in nasal biopsies but not in the peripheral blood after allergen challenge.[13] These data strongly suggest that sIL-2R in the nasal secretion originates from helper T cells in the nasal mucosa.

Eosinophils are the major effector cells in the late response of allergic inflammation causing nonspecific hyperresponsiveness of the mucosa manifested by sustained clinical symptoms of nasal allergy. In the present study, we failed to find a correlation between serum levels of sIL-2R and peripheral blood eosinophil count, or between the levels of sIL-2R in the nasal secretions and graded eosinophilia in nasal smears. However, a significant correlation was found between serum sIL-2R and blood eosinophil count in asthmatic patients.[12] Furthermore, it has also been reported that rIL-2 administration as a part of immunotherapy for a malignant tumour induced marked eosinophilia.[14,15] These data still suggest a positive relationship between IL-2 and eosinophil accumulation.

The mechanism of IL-2 induced eosinophil infiltration may possibly be explained by IL-5 derived from activated T cells.[16,17] IL-5 is a potent and specific eosinophil growth factor,[18] and significantly prolongs eosinophil survival.[19] IL-5 also enhances the expression of adhesion molecules on vascular endothelial cells,[20] and induces chemotaxis[21] and activation of eosinophils.[18] Therefore, $CD25^+$ cells stimulated by IL-2 might induce eosinophil infiltration in the nasal mucosa in conjunction with IL-5 released from mast cells following allergen challenge.

In conclusion, we measured sIL-2R levels in the nasal secretions and sera from nasal allergy patients and healthy control subjects, and found elevated levels of sIL-2R concentration in both the nasal secretions and sera in the patients with nasal allergy. Accumulation of $CD25^+$ cells was found in the allergic nasal mucosa by immunocytochemical examination. These results suggest that T cell activation is induced in nasal allergy, which results in persistent inflammation of the nose.

### References

1. Hansel FK. Cytological diagnosis in respiratory allergy and infection. *Ann Allergy* 1966; **24**: 564–569.
2. Krause HF. Nasal cytology in clinical allergy. In: Krause HF, ed. *Otolaryngologic Allergy and Immunology*. Philadelphia: WB Saunders Company, 1989; 112–122.
3. Linder A, Venge P, Deuschel H. Eosinophil cationic protein and myeloperoxidase in nasal secretions as markers of inflammation in allergic rhinitis. *Allergy* 1987; **42**: 583–590.
4. Smith KA. T-cell growth factor. *Immunol Rev* 1980; **51**: 337–357.
5. Nikaido T, Shimizu A, Ishida N, *et al.* Molecular cloning of cDNA encoding human interleukin-2 receptor. *Nature* 1984; **311**: 631–635.
6. Hatakeyama M, Tsudo M, Minamoto S, *et al.* Interleukin-2 receptor β chain gene: generation of three receptors from by clone human α chain and β chain cDNA's. *Science* 1989; **244**: 551–556.
7. Takeshita T, Asao H, Suzuki J, Sugamura K. An associated molecule, p64, with high affinity interleukin receptor. *Int Immunol* 1990; **2**: 477–480.
8. Rubin LA, Kurman CC, Fritz ME, *et al.* Soluble interleukin-2 receptors are released from activated human lymphoid cells *in vitro*. *J Immunol* 1985; **135**: 3172–3177.
9. Yasuda N, Patric K, Steohen HP, *et al.* Soluble interleukin 2 receptors in sera of Japanese patients with adult T cells. *Blood* 1988; **71**: 1021–1026.
10. Lang BA, Silverman ED, Laxwe RM, Rose V, Nelson DL, Rubin LA. Serum soluble interleukin-2 receptor levels in Kawasaki disease. *J Pediatrics* 1990; **116**: 592–596.
11. Symons JA, Wood NC, DiGiovine FS, *et al.* Soluble IL-2 receptor in rheumatoid arthritis. Correlation with disease activity, IL-1 and IL-2 inhibition. *J Immunol* 1988; **141**: 2612–2618.
12. Lassalle P, Sergant M, Delneste Y, *et al.* Levels of soluble IL-2 receptor in plasma from asthmatics. Correlations with blood eosinophilia, lung function, and corticosteroid therapy. *Clin Exp Immuol* 1992; **87**: 266–271.
13. Verney VN, Jacobson MR, Sudderick RM, *et al.* Immunohistology of the nasal mucosa following allergen-induced rhinitis. Identification of activated T lymphocytes, eosinophils, and neutrophils. *Am Rev Respir Dis* 1992; **146**: 170–176.
14. Kovach JS, Gleich GJ. Eosinophilia and fluid retention in systemic administration of interleukin-2. *J Clin Oncol* 1986; **4**: 815–816.
15. Silberstein DS, Schoof DD, Rodrick ML, *et al.* Activation of eosinophils in cancer patients treated with IL-2 and IL-2-generated lymphokine-activated killer cells. *J Immunol* 1988; **142**: 2162–2167.
16. Enokihara H, Furusawa S, Nakakubo H, *et al.* T cell from eosinophilic patients produce interleukin-5 with interleukin-2 stimulation. *Blood* 1987; **73**: 1809–1813.
17. Yamaguchi Y, Suda T, Shiozaki H, *et al.* Role of IL-5 in IL-2 induced eosinophilia *in vivo* and *in vitro* expression of IL-5 mRNA by IL-2. *J Immunol* 1990; **145**: 873–877.
18. Lopez AF, Sanderson CJ, Gambele JR, Campbell HR, Young IG, Vadas MA. Recombinant human interleukin 5 is a selective activator of human eosinophil function. *J Exp Med* 1988; **167**: 219–224.
19. Yamaguchi Y, Hayashi Y, Sugama Y, *et al.* Highly purified murine interleukin 5 (IL-5) stimulates eosinophil function and prolongs *in vitro* survival. *J Exp Med* 1988; **167**: 1737–1742.
20. Walsh GM, Hartnell A, Wardlaw AJ, *et al.* IL-5 enhances the *in vitro* adhesion of human eosinophils, but not neutrophils, in a leukocyte integrin (CD11/18)-dependent manner. *Immunology* 1990; **71**: 258–265.
21. Wang JM, Rambaldi A, Biondi A, Chen ZG, Sanderson CJ, Mantovani A. Recombinant human interleukin-5 is a selective eosinophil chemoattractant. *Eur J Immunol* 1989; **19**: 701–705.

ACKNOWLEDGEMENT. This study was performed using soluble interleukin-2 receptor assay kits kindly provided from Yamanouchi Pharmaceutical Co. Ltd, Tokyo.

**Received 14 September 1994; accepted in revised form 7 October 1994**
